# Analytical Validation of a Serum Biomarker Signature for Detection of Early-Stage Pancreatic Ductal Adenocarcinoma

**DOI:** 10.3390/diagnostics15243177

**Published:** 2025-12-12

**Authors:** Robyn Pescatore, Naphtali Milliken, Thomas King, Dillon Josey, Norma A. Palma, Lisa Ford

**Affiliations:** Immunovia, Inc., Durham, NC 27713, USA; robyn.pescatore@immunovia.com (R.P.); thomas.king@immunovia.com (T.K.);

**Keywords:** pancreatic cancer, biomarkers, analytical, validation, ELISA

## Abstract

**Background/Objectives**: Early detection of pancreatic ductal adenocarcinoma (PDAC) can improve patient survival and biomarkers to facilitate this are greatly needed. We recently reported a serum biomarker signature comprising tissue inhibitor of metalloproteinase 1 (TIMP1), intercellular adhesion molecule 1 (ICAM1), cathepsin D (CTSD), thrombospondin 1 (TSP1/THBS1), and carbohydrate antigen 19-9 (CA 19-9), that detected Stage I and II PDAC with high sensitivity and specificity. In this assay, CA 19-9 is measured with a commercial instrument and individual ELISAs were developed to measure TIMP1, ICAM1, CTSD, and THBS1. Here, we report the analytical performance of these four analytes in their ELISA formats. **Methods**: Biomarker precision, linearity, algorithm precision, matrix effects, hook effect, method comparison, interference, and analyte stability were evaluated against acceptance criteria per CLSI guidelines. **Results**: High, medium, and low concentrations of each biomarker met acceptance criteria for inter- and intra-day precision (%CVs < 14%) and for linearity (%CVs < 11%). Matrix effects did not impact quantitation of any analyte nor was hook effect present. All analytes met acceptance criteria for accuracy and stability (all biases < 11.2% and <16.5%, respectively). For interference, two CTSD measurements and one ICAM1 measurement in HAMA-spiked samples showed 20.7–29% biases, falling slightly outside of acceptance criteria (<20% bias). All other analyte concentrations met interference acceptance criteria. In total, 94.1% of all diagnostic calls were made with 100% certainty, indicating high precision of the assay’s algorithm. **Conclusions**: All analytes demonstrated acceptable analytical precision, linearity, accuracy, and stability, showing high overall analytical performance of each analyte.

## 1. Introduction

Pancreatic cancer is the third leading cause of cancer death in the United States and one of the few cancers that is becoming more prevalent worldwide [1,2,3]. When diagnosed at Stage IV, pancreatic ductal adenocarcinoma (PDAC), which accounts for 90% of pancreatic cancers [4], has a 5-year survival rate of 3.1% [5]. Survival improves to 44% when diagnosed before metastasis and to 83% when diagnosed at Stage IA [5,6]. To help ensure early diagnosis, surveillance is recommended for individuals at high risk of developing PDAC, yet today’s imaging techniques have limited ability to detect small early-stage tumors [7,8,9], underscoring the importance of identifying biomarkers to aid in surveillance and diagnosis of early-stage disease.

Currently, carbohydrate antigen 19-9 (CA 19-9), the only biomarker approved for use in PDAC patients, can inform the patient’s response to treatment but is precluded from serving as a diagnostic marker due to low sensitivity [10,11]. However, the inclusion of CA 19-9 has been shown to improve diagnostic success of PDAC in diverse biomarker panels [12,13,14,15,16,17,18,19]. Recently, we reported a serum-based biomarker signature comprising tissue inhibitor of metalloproteinases 1 (TIMP1), intercellular adhesion molecule 1 (ICAM1), cathepsin D (CTSD), thrombospondin 1 (THBS1), and CA 19-9, which were identified using machine learning in a model development study aimed at discovering biomarkers to facilitate the diagnosis of early-stage PDAC [20]. In two independent validations that utilized two independent cohorts of Stage I and II PDAC cases and high-risk controls, the signature, which we have termed PancreaSure^TM^, detected PDAC with sensitivities of 78.5% and 76.5% and specificities of 93.5% and 87.8% [21,22]. In both validations the test demonstrated similar performance in clinically relevant sub-populations including those with diabetes, mucinous pancreatic cysts, and ≥65 years of age.

TIMP1, ICAM1, CTSD, and THBS1 each play a role in PDAC pathophysiology and are differentially expressed in PDAC tumors compared to noncancerous tissue. High expression of TIMP1 and ICAM1 is associated with poor prognosis, and both proteins promote metastasis by enhancing neutrophil and macrophage infiltration into the pancreas and liver to thereby facilitate development of a premetastatic niche for tumors [19,23,24]. CTSD and THBS1 are typically over expressed in PDAC, and each protein supports tumor growth and metastasis through diverse mechanisms [25,26,27,28]. Owing to their roles in PDAC progression, each of these analytes has been implicated as a potential diagnostic biomarker for PDAC [15,16,17,18,19], yet to our knowledge, our study is the first to incorporate these analytes into a biomarker signature that detects PDAC with high sensitivity and specificity in Stage I and II cases and in high-risk controls. As part of our efforts to further validate the performance of these analytes, we developed enzyme-linked immunosorbent assays (ELISAs) to measure TIMP1, ICAM1, CTSD, and THBS1 [20]. The goal of this study was to validate the analytical performance of TIMP1, ICAM1, CTSD, and THBS1 in their ELISA formats and evaluate the total precision of the diagnostic algorithm. CA 19-9 was not included in validation because it is measured using a commercial assay and instrument (Elecsys CA 19-9 Immunoassay on COBAS 8000 e602 modular analyzer, Roche Diagnostics, Rotkreuz, Switzerland) that have passed analytical validation studies required for FDA approval [29,30], and was verified according to manufacturer protocols.

## 2. Materials and Methods

### 2.1. Study Design and Patient Demographics

Validation experiments were performed on healthy serum samples (*n* = 30) purchased from Discovery Life Sciences, Inc. (Huntsville, AL, USA) and on leftover volumes of de-identified serum samples that had been retrospectively analyzed in the PancreaSure^TM^ development study [20]. Samples were collected at 12 study sites across the United States according to protocols that adhered to ethical standards outlined in the Declaration of Helsinki and were approved by the Institutional Review Boards/Ethics Committees of all participating sites. All study participants provided written informed consent. Inclusion and exclusion criteria for the study are shown in Supplemental Table S1.

### 2.2. Sample Collection, Processing, and Storage

For patient samples, a minimum of 4 mLs of blood was collected in red-topped serum tubes (Becton Dickinson, Franklin Lakes, NJ, USA #367815), inverted several times, and allowed to clot at room temperature (RT) for 30–60 min. Tubes were then centrifuged at 1300× *g* for 10 min at RT. Serum supernatant was transferred to cryotubes (VWR, Radnor, PA, USA #10026-102) in 250 µL amounts. Tubes were labeled and stored at −80 °C until being shipped to Immunovia frozen on dry ice.

### 2.3. Materials and Reagents

Microtiter plates coated with capture (mouse monoclonal) antibodies for ICAM1 were purchased from Proteomedix AG (Schlieren, Switzerland, MTP-ICAM1). Microtiter plates coated with capture (mouse monoclonal) antibodies for CTSD and THBS1 were supplied by MediagnostGmbH (Reutlingen, Germany, MTP-CTSD, MTP-THBS1). Biotinylated detection solutions for CTSD, ICAM1, and THBS1 (DET, containing goat polyclonal antibodies) were supplied by MediagnostGmbH. Mouse monoclonal TIMP1 capture antibody (#MAB970) and goat polyclonal detection antibody (#BAF970) were purchased from Bio-techne (Minneapolis, MN, USA). Interference test kits containing hemoglobin, free and conjugated bilirubin, triglycerides (#K2010001), and biotin (K201008) were purchased from Molecular Depot (San Diego, CA, USA). HAMA interference kits were purchased from Molecular Depot (#K2010012). For calibration standards and QC samples, TIMP1 protein was purchased from Bio-techne (#970-TM-010), ICAM1 protein was purchased from Proteomedix AG (#ICAM1 PROT), and CTSD and THBS1 proteins were supplied by MediagnostGmbH (CAL-CTSD, CAL-THBS1).

### 2.4. Biomarker Measurements

All protein biomarkers were measured using individual ELISA materials according to previously developed protocols [20]. For TIMP1, briefly, plates were manually coated with capture antibody at 4 °C overnight, washed once with PBST (IBI Scientific, Dubuque, IA, USA #1B70168-10) then blocked with blocking solution (Boca Scientific, Dedham, MA, USA, #110-902) at RT for 1 h. For every run, samples and controls were diluted with LowCross Buffer^®^ (Boca Scientific, #100-902) as follows: 1:164 for TIMP, 1:41 for ICAM1 and CTSD, and 1:1681 for THBS1. Detection solutions for each biomarker were prepared by adding an experimentally derived amount of the biomarker-specific detection antibody to 6.5 mL of blocking solution. HRP solution for ICAM1 and TIMP 1 was prepared by adding 2.5 µL of Ultra Streptavidin-HRP (Thermo Fisher Scientific, Waltham, MA, USA, #N504) to 25 mL of HRP-Protector (Boca Scientific, # 222-902). HRP solution for THBS1 and CTSD was provided by MediagnostGmbH. TMB substrate solution for THBS1 and CTSD (MediadiagnostGmbH, Reutlingen, Germany #PTX TMB), and for TIMP 1 and ICAM1 (Neogen, Lansing, MI, USA, #308177) was used to develop color and 0.25 M sulfuric acid (Lab Chem, Zelienople, PA, USA #LC257402) was used to stop the colorimetric reaction. Diluted samples, detection reagents, and color and stop solutions were vortexed thoroughly then added to a Dynex DSX Automated ELISA Analyzer (Dynex Technologies, Chantilly, VA, USA) where the remaining assay steps were performed. Plates were incubated with detection solution and samples for 1 h, washed 3 times with PBST, then incubated with HRP solution for 30 min. After 3 washes with PBST, TMB substrate solution was added for 30 min followed by stop solution. Absorbance was measured at 450 nm with reference wavelength 620 nm. For all experiments except linearity of calibration, which used undiluted samples, values were calculated by multiplying the absorbance at 450 nm by the dilution factor for each analyte.

### 2.5. Analytical Performance and Acceptance Criteria

#### 2.5.1. Analytical Sensitivity

Dilutions of low concentration samples that approached the expected limit of detection (LOD) were run over at least 3 days with at least 60 total replicates per sample. The lower limit of quantitation (LLOQ) was defined as the lowest measurement on the calibration curve and was tested by including the lowest calibration standard in quadruplicate in each run conducted over at least 3 days with at least 60 total replicates. The limit of the blank (LOB) was determined by multiple measurements of the blank totaling at least 60 replicates. The LOB was calculated as shown.

(1) $$LOB=Mean\;of\;the\;blank+1.65\;\left(standard\;deviation\right)$$

Due to the shape of the 4-parameter logistic curve generated by the model used in these tests, the protein concentrations of the blank and LOD samples tended to approach the curve’s lower asymptote and could not always be calculated. For this reason, the LOB and LOD were determined based on absorbance rather than protein concentration. Those absorbance values were compared to the average absorbance of the LLOQ. The LOD was acceptable if its absorbance was less than the LLOQ’s absorbance. Signal-to-noise (S/N) was also calculated by dividing the inter-day mean response of the LLOQ and LOD by the inter-day blank mean response.

#### 2.5.2. Linearity of Calibration

The clinical and analytical measurement range of each protein was determined in the model development study [20]. We note that since the outcome of the test is binary, we report measurement ranges, rather than reference ranges, for individual analytes. Five dilutions spanning the measurement range, which included the LLOQ, were run in quadruplicate, and the accuracy of calibration sample measurements were calculated as shown.

(2) $$\%\;Accuracy=\left(\frac{Measured\;concentration}{Expected\;concentration}\right)\times 100$$

Acceptance criteria for the LLOQ was +/−25% of its expected concentration. Criteria for the other 4 dilutions was +/−20% of their expected concentrations.

#### 2.5.3. Biomarker Precision

Precision for each biomarker in serum was measured at the upper, medium, and lower ends of the measurement range using individual QC aliquots. Precision was also measured at the LLOQ of each analyte. 4 replicates of each concentration were measured daily for 20 days (*n* ≥ 80). Replicates were made from individual aliquots that were divided at the beginning of the study and stored at −80 °C until their run time. Precision was deemed acceptable for high, medium, and low samples if their inter- and intra-day % coefficients of variation (%CVs) ≤ 15%. Precision of the LLOQ was deemed acceptable if its inter- and intra-day %CV ≤ 20%. %CV was calculated as shown.

(3) $$\%CV=\left(\frac{Replicate\;Standard\;Deviation}{Replicate\;Avg\;Measurement}\right)\times 100$$

#### 2.5.4. Reportable Range, Dilutional Linearity, and Hook Effect

Linearity was evaluated using patient samples that were serially diluted to span each analyte’s reportable range. Three dilution series for each analyte included one concentration that exceeded the upper limit of quantitation (ULOQ) by 5–129%. These high-concentration samples were used to evaluate hook effect. Four replicates of each dilution were analyzed. Intra-dilution linearity was considered acceptable if %CV ≤ 15%. Hook effect was considered absent if absorbance was maintained when analyte concentrations exceeded the ULOQ.

#### 2.5.5. Accuracy

During the biomarker discovery [31] and signature development studies [20] the proteins TIMP1, ICAM1, CTSD, and THBS1 were measured in clinical samples by Proteomedix AG (Schlieren, Switzerland) using single ELISAs performed according to proprietary protocols. Accuracy of the method used in this validation was evaluated by comparing the amount of each protein measured in-house at Immunovia to previously obtained measurements by Proteomedix AG. This approach, rather than a traditional head-to-head comparison between methods was taken because there are currently no analytically validated methods for quantitating TIMP1, ICAM1, CTSD, or THBS1, and the overall goal was to identically match the quantitation of the assay as used in the model development study. Sixteen patient samples with concentrations that had been established over multiple runs at Proteomedix AG were analyzed in a single run at Immunovia. These particular samples were chosen from the ones available because they covered the reportable range as much as possible. Inter-series effects were controlled by internal QC samples. Accuracy was considered acceptable if biases between Proteomedix and Immunovia measurements were < 20%. Correlation between measurements was also evaluated.

#### 2.5.6. Stability

Analyte stability was evaluated at room temperature (RT), 4 °C, and after 3 and 5 freeze/thaw cycles. All experiments were performed on 4 replicates of low, medium, and high QCs. Aliquots undergoing RT or 4 °C stability were removed from −80 °C and left at either RT (19.2–20.3 °C) or 4 °C for 24, 48, and 72 h. An additional experiment was performed to evaluate the stability of CTSD after 8.5 h at RT. For freeze/thaw analyses aliquots were removed from −80 °C, thawed to completion, then returned to the freezer. Average measurements of each replicate were compared to averaged measurements from freshly thawed replicates. Acceptance criteria was <20% bias between baseline and comparator samples.

#### 2.5.7. Analytical Specificity

We determined whether analyte quantitation was affected by interference with hemoglobin, triglycerides, bilirubin, biotin, proteins closely related to the analytes, and mouse anti-human antibodies (HAMA). Biotin was included in this analysis because it is a common nutritional supplement that may interfere with the biotinylated antibodies used in the ELISA protocols. In accordance with CLSI guidelines [32], 4 replicates of high- and low-concentration patient samples or QCs of each analyte were spiked with each substance to the following final concentrations: (1) 1000 and 300 mg/dL hemoglobin, (2) 40 mg/dL conjugated and unconjugated bilirubin, and (3) 1500 mg/dL triglycerides. Biotin was spiked to a final concentration of 100 ng/mL, the upper limit of the COBAS instrument used to measure it. Hemoglobin interference was initially tested with 1000 mg/dL but then re-tested at 300 mg/dL for results initially outside of acceptance criteria. Closely related proteins were spiked into samples at twice the highest concentration on the calibration curve: tissue inhibitor of metalloproteinase 2 (TIMP2, 50 ug/mL) (Bio-techne, #971-TM-010), intercellular adhesion molecule 4 (ICAM4, 20 ng/mL) (Bio-techne, #10407-IC-050), Cathepsin B (CTSB, 5 ug/mL) (Bio-techne, #953-CY-010), and thrombospondin 2 (THBS2, 50 ug/mL) (Bio-techne, #1635-T2-050). For HAMA experiments, 9 HAMA-containing samples from the kit were spiked with HAMA blocker at 50:1 *v*/*v* according to the manufacturer’s instructions and compared to un-spiked samples. All un-spiked samples were prepared using diluent volumes equal to the total volume of spiked samples. For all interference experiments, analyte concentrations in spiked samples were compared to concentrations in un-spiked samples. Acceptance criteria was <20% bias between spiked and un-spiked sample measurements. % bias was calculated as shown.

(4) % bias=((Spiked−Unspiked)/Unspiked))×100

#### 2.5.8. Algorithm Precision

The total precision of the algorithm was modeled using a Monte-Carlo simulation in which the %CVs of the analytes during validation were used to simulate random permutations of results from all clinical sample results from our model development study [20]. For each sample, the measured values of CA19-9, TIMP1, ICAM1, CTSD, and THBS1 were used in conjunction with the analytically validated %CV for each marker (the percent amount that measurement is expected to vary on repeat testing). Using these two inputs, 1000 simulated measurement sets of the same sample were created by shifting each biomarker value randomly upward or downward in proportion to its %CV, representing the range of values expected from normal assay variation. The algorithm score was then recalculated for each of these 1000 generated versions and compared with the fixed algorithm score cutoff used to determine a binary positive/negative result. Confidence was defined as the proportion of generated versions that produced the same classification as the original score. Diagnostic uncertainty was defined as the proportion that produced a score on the opposite side of the cutoff. The purpose of the Monte-Carlo simulation was to estimate the analytical uncertainty and subsequent reliability of the decision score for individual samples. It was not used for algorithm calibration or to evaluate diagnostic accuracy.

### 2.6. Statistical Analyses

Calibration curves were generated by applying 4-parameter logistical regression to absorbance values and multiplying the computed concentration of the analyte by its dilution factor. All measurements were performed in duplicate using 3 independently prepared dilutions. Plates were accepted and the individual experiments evaluated if (1) the signal of the highest calibrator was above 1.0 and the lowest calibrator below 0.2, (2) the R^2^ value of the calibration curve was greater than 0.95, and (3) the back-calculated value of the standards against the calibration curve was within 15% of the nominal value. All statistical analyses were performed in Microsoft Excel and REVELATION DSX^®^ software version 6.40.256.10058 (Dynex Technologies). All reagents were validated across lots before experimental runs.

## 3. Results

### 3.1. Analytical Sensitivity

The LOD, LOB, and LLOQ were calculated for each analyte (Supplemental Table S2). The signal to noise of the LOD and LLOQ were also calculated. For CTSD, TIMP1, and THBS1, the calculated LOD was less than the average absorbance of the LLOQ, thus meeting acceptance criteria. The absorbance of the LOD and LLOQ were similar in magnitude for ICAM1, as were the S/N of ICAM1 at the LOD and LLOQ. The average LLOQ S/N was > 4 for all four proteins.

To further examine the potential error caused by the ICAM variability at the LLOQ, we examined the contribution of this potential error to the scores from the model development study [20]. Out of 738 samples used in the study, 89 (12%) had ICAM results lower than the low QC. For these 89 samples, the score incorporating this error for ICAM in the positive and negative direction was calculated and compared to the original scores. Only two samples experienced a shift that changed their binary determination. Both of these were from negative to positive when the ICAM was varied in the positive direction, and no samples shifted from positive to negative. For the two samples in question, the original ICAM values were within 10% of the low QC.

### 3.2. Linearity of Calibration

Representative calibration curves are shown in Supplemental Figure S1. All analytes met acceptance criteria in every run by achieving 100% accuracy +/− 25% (LLOQ) or +/−20% (the other four dilutions) with one exception. In run 18, the sample with the lowest concentration of ICAM1 demonstrated 70.9% accuracy (Supplemental Tables S3–S6).

### 3.3. Biomarker Precision

The precision of each analyte was measured at the high, medium, low levels of each measurement range, and at the LLOQs. High, medium, and low concentrations of every analyte met acceptance criteria for inter- and intra-day precision with all %CV < 14% (Table 1). LLOQ samples of TIMP1, CTSD, and THBS1 also demonstrated acceptable inter- and intra-day precision. Although the average intra-day precision of ICAM1’s LLOQ was acceptable, its inter-day precision fell outside of acceptance criteria, achieving a %CV of 36.7%. Analyte concentrations and individual %CVs for each measurement are shown in Supplemental Tables S7–S10.

### 3.4. Reportable Range, Dilutional Linearity, and Hook Effect

**Figure 1:**
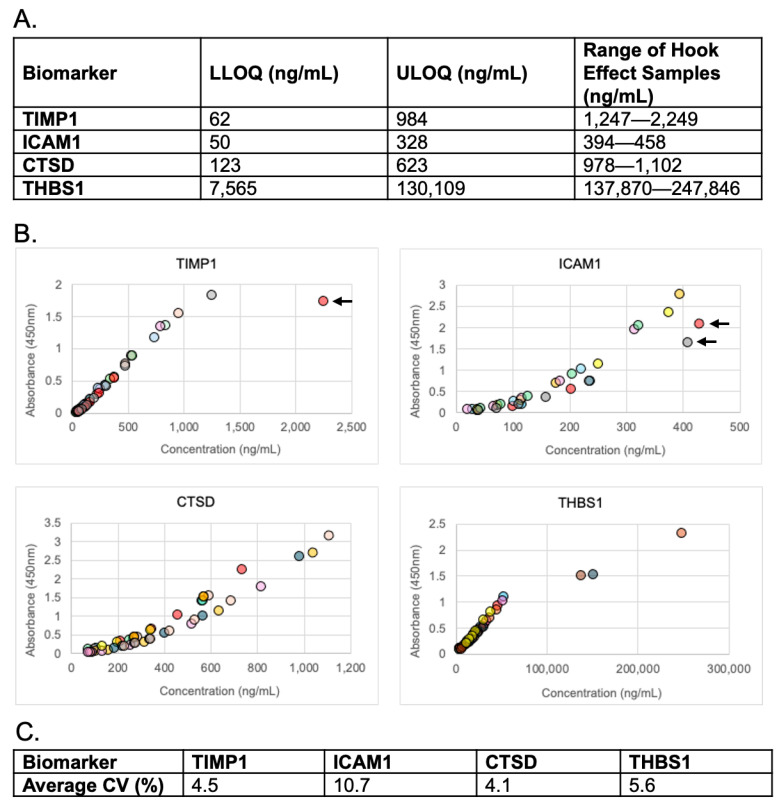
**Linearity and hook effect.** (**A**) Reportable range of each analyte and ranges of high concentration samples used to evaluate hook effect. LLOQ = lower limit of quantitation, ULOQ = upper limit of quantitation. (**B**) Serial dilutions of patient samples. Arrows indicate high concentrations of response curves that were lower than the other curves in that analyte group. (**C**) Average % CVs for each analyte’s intra-dilutional linearity.

To evaluate linearity, we analyzed nine patient samples that were serial diluted according to each analyte’s reportable range (Figure 1A). Linearity was maintained for each analyte measurement (Figure 1B). Absorbance of high-concentration (i.e., hook effect) samples demonstrated increased absorbance in response to the elevated concentration. Although one high-concentration sample for TIMP1 (Figure 1B, TIMP1 graph, arrow) and two high concentration samples for ICAM1 (Figure 1B, ICAM1 graph, arrows) appeared to decrease, the response curve was lower for those dilutions on the day they were run vs. other days, thus the data ultimately show that hook effect was not present up to the highest concentration tested. The average intra-dilutional linearity %CVs for all analytes was < 10.7% (Figure 1C). %CVs for each dilution sample met acceptance criteria except two ICAM1 dilutions, which demonstrated %CVs of 18.7% and 24.1% (Supplemental Table S11).

### 3.5. Accuracy

**Figure 2:**
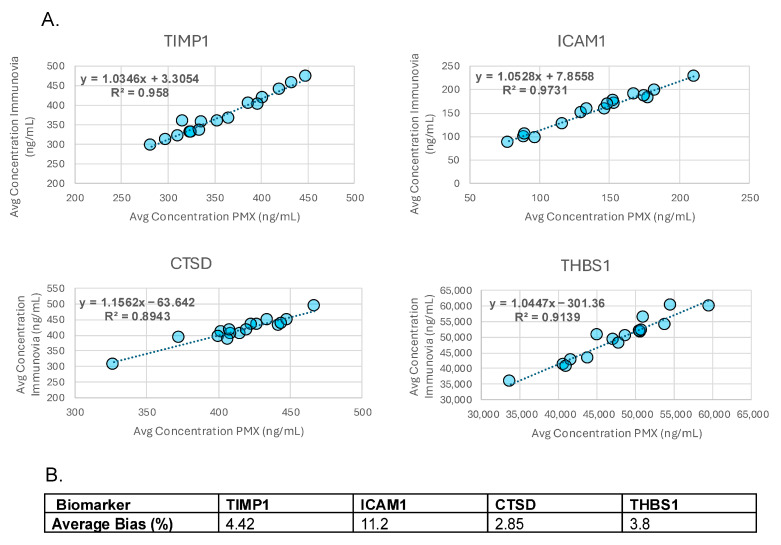
**Accuracy**. (**A**) Graphs showing correlation (R^2^) between analyte measurements performed on the same samples by Immunovia and Proteomedix AG (PMX). (**B**) Average % biases for each analyte.

Accuracy was determined by comparing measurements taken from the same patient samples by two different laboratories. Measurements showed good correlation with all R^2^ values > 0.89 (Figure 2A). Both individual and average % biases for each analyte met acceptance criteria (Figure 2B and Supplemental Table S12).

### 3.6. Stability

TIMP1, ICAM1, and THBS1 demonstrated acceptable stability for up to 72 h at room temperature (RT). CTSD did not meet acceptance criteria for RT stability with the exception of high concentration samples at 24 h (Table 2). To better characterize the stability of CTSD, we repeated the analysis at RT for 8.5 h. Under these conditions, all concentrations of this analyte met acceptance criteria (Table 2).

All analytes remained stable in response to five freeze/thaw cycles (Table 3). Long-term analyte stability was evaluated in PancreaSure’s clinical validation on samples that had been stored at −80 °C for 1–10 years [21]. The diagnostic sensitivity and specificity was highest on samples stored for ≤5 years. By contrast, there was a significant decline in test performance in samples that had been in storage for >5 years.

### 3.7. Analytical Specificity

We evaluated specificity by determining the impact of interfering substances on analyte quantitation. All specificity experiments met acceptance criteria, with the exception of a single ICAM1 sample and two CTSD samples spiked with HAMA, which demonstrated biases of 20.7% (ICAM1), and 28.79%, and 22.07% (CTSD). The low concentration of CTSD spiked with 1000 mg/dL hemoglobin fell outside acceptance criteria (−25.3% bias) but met acceptance criteria when spiked with 300 mg/dL hemoglobin (Table 4).

The potential error caused by the maximum CTSD HAMA interference (28.79%) was calculated on the model development study data [20]. The number of samples that crossed the cutoff from negative to positive when the amount of CTSD was increased by 28.79% was determined to be 9 out of 738 total samples, or 1.2%.

### 3.8. Algorithm Precision

**Figure 3:**
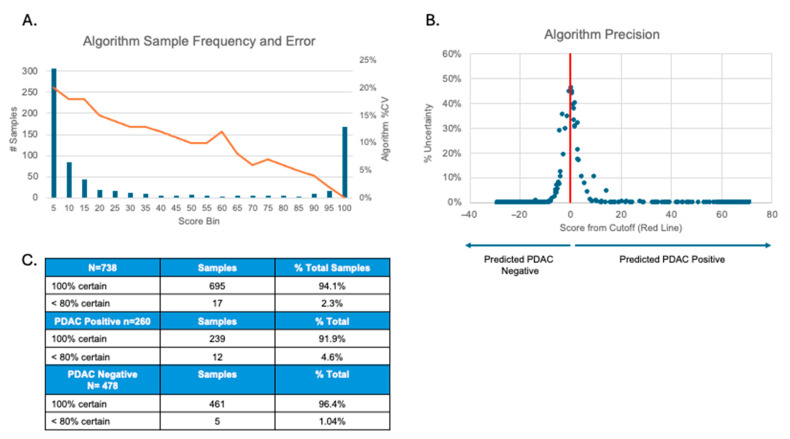
**Algorithm precision**. (**A**) Graph showing the %CV range (0–20%) of algorithm scores. (**B**) Graph showing the percentage of samples that were called with less than 100% certainty in relation to algorithm score. The cutoff score is shown as 0 on the *x*-axis. *X*-axis numbers are arbitrary units showing the positive or negative change in algorithm score relative to the cutoff. (**C**) The number of positive, negative, and total simulations that were called with the indicated certainties.

A Monte-Carlo simulation showed that the total %CV of the algorithm varied inversely by score, from 0 to 20% (Figure 3A). Diagnostic certainty was highest at the extremes of scoring and decreased as the score approached the cutoff (Figure 3B). Notably, 695 out of 738 sample determinations (94.1%) exhibited 100% certainty, while only 17 (2.3%) were called with <80% certainty. Similar results were observed when PDAC positive and PDAC negative calls were analyzed as separate groups. Here, 91.9% of positives and 96.4% of negatives achieved 100% certainty (Figure 3C).

## 4. Discussion

In the absence of a curative treatment for late-stage PDAC, diagnosing this malignancy in its early stages is crucial to improving patient survival rates. Implementing non-invasive biomarkers in disease surveillance alongside today’s standard of care imaging technologies could help maximize early-stage diagnostic success. Here, we report the analytical performance of individual ELISAs that measure TIMP1, ICAM1, CTSD, and THBS1, four analytes in a serum biomarker signature that previously demonstrated high sensitivity and specificity in detecting Stage I and II PDAC [20,21,22].

Each ELISA demonstrated acceptable inter- and intra-day precision at the high, medium, and low ends of each analyte’s reportable range. TIMP1, CTSD, and THBS1 demonstrated acceptable inter- and intra-day precision at their LLOQs. ICAM1 demonstrated acceptable average intra-day precision at its LLOQ, but its inter-day precision fell outside of acceptance criteria. Analytical sensitivity experiments also showed that the ICAM1 LLOQ is approximately equivalent to the LOD, which is likely a contributing factor in the LLOQ imprecision. Further evaluation of samples with ICAM1 levels below the low QC showed that when the original test results of these samples were compared to results that incorporated this error for ICAM1 in the positive or negative direction, only two samples experienced a shift that changed their score. For these two samples, the original ICAM values were actually within 10% of the low QC, which also implies that the actual error in the score would be more similar to the low QC. When the error of the low QC is used, the values still do cross the cutoff from negative to positive, implying that the higher error at the LLOQ has little impact beyond what has been stated in the Monte-Carlo analysis of the full data set (Figure 3). Subsequent clinical validation studies have shown that the inter-day precision at the LLOQ for ICAM1 does not have a significant effect on the final determination of the test [21,22].

Dilutional linearity was analyzed to determine whether diluted sample measurements could be expected to linearly and reproducibly span the measurable range of the assay, indicating lack of matrix effect, and to show that different dilution factors can accurately provide quantitation. The variation in intra-dilutional linearity for TIMP1, CTSD, and THBS1 was <14%, showing that accurate measurements can be maintained through their reportable ranges. In seven out of the nine patients, the intra-dilutional linearity of ICAM1 met acceptance criteria. In the two remaining patients, the dilution series for ICAM slightly exceeded acceptance criteria for %CV. Although this may indicate matrix effect, in practice, the samples in this assay are sampled directly from serum and do not require pre-dilution before the assay. Thus, the imprecision of ICAM is more strongly represented by the QC precision experiment.

In hook effect experiments, analyte absorbance continued to linearly increase as the concentration increased to levels that exceeded the ULOQ of the assays. Although a single high-concentration sample of TIMP1 and two high-concentration samples of ICAM1 appeared to decrease with increasing analyte levels, those patient samples demonstrated a lower response curve due to variability in the daily intensity response of the assay. Overall, the results indicate that hook effect was not present.

In method comparison experiments, the relative difference between measurements performed by two different laboratories was ≤11.2% for each analyte, which further demonstrates the ability of each ELISA to accurately quantitate each biomarker according to their intended use. Each analyte also demonstrated acceptable analytical stability at 4 °C for up to 72 h and in response to three and five freeze/thaw cycles (all biases < 20%). TIMP1, ICAM1, and THBS1 remained stable for up to 72 h at RT. Only the high concentration of CTSD remained stable for 24 h at RT, with the low concentration sample being just outside of acceptance criteria. All other CTSD samples left at RT fell outside of acceptance criteria. We evaluated the stability of high, medium, and low concentrations of CTSD after 8.5 h at RT, and, under these conditions, all concentrations of CTSD met acceptance criteria. Given that CTSD remains stable at RT for this length of time, we are confident that the stability of CTSD will not compromise test results under typical analysis conditions in which patient samples are exposed to RT for less than 90 min (see Section 2, Materials and Methods, Sample Collection, Processing, and Storage). During real-world application of this test, samples will be collected and shipped using a supplied kit which contains a NanoCool™ shipping device (Peli BioThermal, Maple Grove, MN USA), and samples arriving at room temperature will be rejected. Additionally, samples that are collected and have the potential to have been at room temperature for more than six hours will also be rejected.

Substances that interfere with assay measurements may result in erroneous diagnostic results. We evaluated the impact of hemoglobin, bilirubin, triglycerides, biotin, and closely related proteins on quantification of analytes at the upper and lower ends of their reportable ranges. We also evaluated interference by HAMA on kit-supplied serum samples. A single ICAM1 measurement from a sample spiked with HAMA blocker fell slightly outside acceptance criteria (20.7% bias), as did two CTSD measurements on HAMA blocker-spiked samples (28.79% and 22.07% biases). Although these samples are outside of acceptance criteria, these results show that the effect of HAMA is not extreme and would be unlikely to affect the final diagnostic determination. We tested this hypothesis on the model development study data and found that 9 out of 738 samples (1.2%) crossed the assay cutoff from negative to positive when the measured amount of CTSD was increased by 28.79%. The number of individuals affected by HAMAs is not precisely known, but even at the higher estimates of 10–40% [33], the number of results affected by the HAMA interference on this assay would still be less than 1%. In total, 1000 mg/dL hemoglobin demonstrated interference outside of acceptance criteria at the low concentration level of CTSD but met acceptance criteria when spiked to 300 mg/dL. It should be noted that sample contamination by hemoglobin at 1000 mg/dL is unlikely if serum isolation and sample preparation are conducted according to standard laboratory protocols. Samples showing elevated levels of hemolysis will ultimately be rejected by the laboratory before processing.

Because actual sample results can contain many permutations of low, medium, and high levels of analytes, the total precision of the algorithm was evaluated using Monte-Carlo modeling of actual samples. The modeling showed that the total %CV of the algorithm varies inversely by score, from 0 to 20%. However, this precision only affects the result in a very narrow area around the cutoff, and in this area, the % CV is approximately 10–15%. The percent uncertainty, which is equivalent to the percentage of times that the score crossed the cutoff in the 1000 Monte-Carlo simulations, is shown in Figure 3. Less than 10% of the samples had any uncertainty at all, and the score was greater than 80% certain for more than 97% of the samples tested. This analysis showed that the score-based classification is highly stable relative to the observed assay variation, with uncertainty confined to samples whose scores lie near the cutoff.

## 5. Conclusions

Overall, the ELISAs were shown to be suitable for quantifying TIMP1, ICAM1, CTSD, and THBS1 in human serum of patients at risk for early-stage PDAC. Their analytical performance is largely unaffected by practices that reflect routine sample handling, storage, and decentralized testing. Furthermore, they demonstrated acceptable performance over time and over the reportable ranges of each analyte. They also demonstrated acceptable performance as part of the full algorithmic result. Taken together, our findings show that the analytes deliver reliable results that can aid in the diagnosis of early-stage PDAC.

## Figures and Tables

**Table 1:** Biomarker precision.

Marker	Sample	Average Concentration (ng/mL) ± SD	Inter-Day CV (%)	Average Intra-Day CV (%)
**TIMP1**	High	833.0 ± 41.5	5.0	3.5
	Medium	330.0 ± 18.3	5.6	3.2
	Low	156.0 ± 13.0	8.3	4.5
	LLOQ	70.6 ± 11.0	15.6	3.7

**ICAM1**	High	279.0 ± 19.7	7.1	5.6
	Medium	152.0 ± 21.2	14.0	6.3
	Low	120.0 ± 13.1	10.9	7.5
	LLOQ	30.2 ± 11.1	36.7	17.5

**CTSD**	High	455.0 ± 20.6	4.5	2.8
	Medium	451.0 ± 29.2	6.5	5.5
	Low	265.0 ± 26.6	10.0	6.7
	LLOQ	180.0 ± 19.7	11.0	5.7

**THBS1**	High	53,811.0 ± 3033.0	5.6	2.7
	Medium	42,014.0 ± 2728.0	6.5	3.2
	Low	27,165.0 ± 2219.0	8.2	4.2
	LLOQ	11,059.0 ± 856.0	7.7	3.0

LLOQ = lower limit of quantitation.

**Table 2:** Analyte stability at room temperature and 4 °C.

	Room Temperature (Average % Bias)								
	24 h			48 h			72 h		
	High	Medium	Low	High	Medium	Low	High	Medium	Low
TIMP1	−11.4	−7.5	−5.8	−18.9	−9.1	−10.0	−18.9	−11.3	−12.5
ICAM1	1.6	−4.7	−5.2	−3.0	−6.3	−1.0	−8.0	−1.1	−3.9
CTSD	−14.6	−22.3	−20.7	−23.4	−39.5	−24.4	−28.5	−50.1	−30.9
THBS1	−5.3	−9.4	−0.4	−9.6	−9.9	−2.4	−12.7	−4.9	−11.6
	8.5 h								
	High	Medium	Low						
CTSD	−11.7	−13.0	−12.0						

	4 °C (average % bias)								
	24 h			48 h			72 h		
	High	Medium	Low	High	Medium	Low	High	Medium	Low
TIMP1	−8.3	−3.4	−7.8	−6.9	−4.7	−10.3	−12.9	−10.3	−10.1
ICAM1	−1.2	−2.3	−4.3	−2.5	−3.7	−3.1	−6.5	−3.4	−1.1
CTSD	−0.7	−7.5	−8.3	−6.1	−6.3	−6.5	−3.9	−13.1	−17.1
THBS1	−6.3	−5.8	−4.6	−5.5	−7.9	1.0	−13.6	−6.6	−3.0

**Table 3:** Analyte stability in response to freeze/thaw (F/T) cycling.

	3 F/T Cycles (Average % Bias)			5 F/T Cycles (Average % Bias)		
	High	Medium	Low	High	Medium	Low
TIMP1	−3.0	−5.8	−0.5	−7.0	−4.8	−4.3
ICAM1	−1.1	−7.1	−4.3	−1.4	−12.2	−4.1
CTSD	−5.7	−5.6	−10.4	−5.5	−13.8	−16.5
THBS1	−4.8	−3.7	−3.2	−4.6	−11.2	−1.3

**Table 4:** Analytical specificity showing % biases between un-spiked and spiked samples.

			Bilirubin				Closely Related Proteins	HAMA	
		**Hemoglobin**	**Conjugated**	**Unconjugated**	**Triglycerides**	**Biotin**	**TIMP 2**	Sample 1	3.2
**TIMP 1**	High Conc.	−1.60	−0.90	5.70	1.90	−5.30	2.2	Sample 2	3.5
	Low Conc.	−8.90	7.60	1.20	8.00	0.80	6.9	Sample 3	1.8
							**ICAM 4**	Sample 1	8.13
**ICAM 1**	High Conc.	−7.10	−6.60	9.60	15.10	−1.90	2.1	Sample 2	15.3
	Low Conc.	1.50	−1.50	8.90	−9.90	−6.70	11.1	Sample 3	20.7
							**CTSB**	Sample 1	−3.10
**CTSD**	High Conc.	−10.30	−3.70	−5.10	−4.50	0.80	−1.55	Sample 2	28.8
	Low Conc.	−7.6	−3.10	3.60	19.70	15.60	4.5	Sample 3	22.1
							**THBS 2**	Sample 1	−0.2
**THBS1**	High Conc.	1.70	4.60	−4.80	3.60	−0.70	6.0	Sample 2	7.3
	Low Conc.	−6.40	−3.10	7.40	5.50	1.30	2.5	Sample 3	1.0

Conc. = concentration.

## Data Availability

The original contributions presented in this study are included in the article/Supplementary Materials. Further inquiries can be directed to the corresponding author.

## Supplementary Materials

The following supporting information can be downloaded at: https://www.mdpi.com/article/10.3390/diagnostics15243177/s1, Figure S1: Representative calibration curves for each analyte showing 4-parameter logistic fit; Table S1: Study inclusion/exclusion criteria; Table S2: Analytical Sensitivity s; Table S3: Linearity of TIMP1 calibration samples; Table S4: Linearity of ICAM1 calibration samples; Table S5: Linearity of CTSD calibration samples; Table S6: Linearity of THBS1 calibration samples; Table S7: TIMP1 Precision; Table S8: ICAM1 Precision; Table S9: CTSD Precision; Table S10: THBS1 Precision; Table S11: %CVs for intra-dilutional linearity measurements; Table S12: Accuracy.

## Abbreviations

The following abbreviations are used in this manuscript:

PDAC	Pancreatic Ductal Adenocarcinoma
TIMP1	Tissue inhibitor of metalloproteinase 1
ICAM1	Intercellular adhesion molecule 1
CTSD	Cathepsin D
THBS1	Thrombospondin 1
CA 19-9	Carbohydrate antigen 19-9
ELISA	Enzyme-linked immunosorbent assay
CLSI	Clinical and Laboratory Standards Institute
LLOQ	Lower limit of quantitation
ULOQ	Upper limit of quantitation
CV	Coefficient of variation
F/T	Freeze/thaw
