# Supplementary material for: Analytical Validation of a Serum Biomarker Signature for Detection of Early-Stage Pancreatic Ductal Adenocarcinoma
Source: Diagnostics (Basel). 2025 Dec 12;15(24):3177. doi: 10.3390/diagnostics15243177 (PMC12731796; doi:10.3390/diagnostics15243177)
Supplement: Supplementary file 1 [file diagnostics-15-03177-s001.zip › Supplemental Table S1.pdf]

Supplemental Table S1. Study inclusion/exclusion criteria.

**Inclusion Criteria**	**Exclusion Criteria**
• ≥ 40 years of age<br>• Recent diagnosis of Stage I or Stage II PDAC<br>or<br>• Participant in high-risk surveillance programs for PDAC in the United States	• Any prior treatment for PDAC including but not limited to prior resection, radiotherapy, or chemotherapy<br>• Current immunosuppressive therapy<br>• Major surgery or significant trauma within 12 weeks prior to blood sample collection<br>• Reported chronic pancreatitis<br>• Co-occurring malignancies diagnosed ≤ 3 years prior to sample collection<br>• Controls reported as symptomatic for disorders, other than diabetes, that may elevate CA 19-9 unrelated to PDAC (e.g., chronic pancreatitis, malignancies, obstructive GI abnormalities, high-grade dysplasia, mucinous cystic neoplasm of the pancreas, etc.)<br>• Sample volume insufficient for analysis
