# Supplementary material for: Analytical Validation of a Serum Biomarker Signature for Detection of Early-Stage Pancreatic Ductal Adenocarcinoma
Source: Diagnostics (Basel). 2025 Dec 12;15(24):3177. doi: 10.3390/diagnostics15243177 (PMC12731796; doi:10.3390/diagnostics15243177)
Supplement: Supplementary file 1 [file diagnostics-15-03177-s001.zip › Supplemental Table S10.pdf]

Supplemental Table S10. THBS1 Precision. Concentrations and %CVs for individual measurements of THBS1.

Run ID	Concentration Level	Concentration (ng/mL) * dilution factor (1641)				Intra-day Average	Intra-day SD	Intra-day %CV	Run ID	Concentration Level	Concentration (ng/mL) * dilution factor (1641)				Intra-day Average	Intra-day SD	Intra-day %CV
1	High	62371	60763	53298	54222	57664	4570.4	7.93	11	High	55867	58898	57808	57989	57641	1275.0	2.21
	Median	42271	43443	43011	43381	43026	538.3	1.25		Median	45184	47120	44173	44701	45294	1284.9	2.84
	Low	26184	27565	28755	29005	27878	1291.9	4.63		Low	30772	30680	28721	28721	29723	1158.4	3.90
2	High	50816	50438	52222	52601	51519	1052.8	2.04	12	High	56441	52674	52809	52493	53604	1895.6	3.54
	Median	40174	42454	41261	40392	41070	1034.9	2.52		Median	41494	39105	38090	36944	38908	1936.5	4.98
	Low	26439	25106	28280	28103	26982	1500.7	5.56		Low	26537	26932	25220	25001	25923	955.6	3.69
3	High	52156	51336	49862	50353	50927	1023.5	2.01	13	High	49700	51018	47409	46247	48594	2161.4	4.45
	Median	41697	42083	37642	39316	40184	2090.5	5.20		Median	38552	39504	37918	37376	38337	914.4	2.39
	Low	26253	25206	24458	24957	25219	756.7	3.00		Low	21489	22027	24755	24710	23245	1731.2	7.45
4	High	54248	53906	52205	52827	53297	946.9	1.78	14	High	55001	53317	52501	52991	53453	1085.3	2.03
	Median	42861	43024	41663	42207	42439	626.2	1.48		Median	42666	41138	40455	41024	41321	945.3	2.29
	Low	25230	24580	27609	27781	26300	1634.0	6.21		Low	25227	25294	20708	21214	23111	2490.9	10.78
5	High	55435	54943	55374	53960	54928	681.7	1.24	15	High	55431	54375	50263	47758	51957	3578.4	6.89
	Median	44235	43280	42088	42029	42908	1056.1	2.46		Median	43336	43954	41870	40908	42517	1383.9	3.25
	Low	25691	24751	29634	29634	27427	2576.6	9.39		Low	33282	31375	28576	27459	30173	2647.2	8.77
6	High	55959	54828	52807	52416	54002	1678.8	3.11	16	High	56581	55526	56965	55909	56245	648.5	1.15
	Median	44256	43655	41307	41525	42686	1489.3	3.49		Median	44337	45421	43442	43536	44184	917.2	2.08
	Low	27605	26495	27721	27953	27444	648.6	2.36		Low	29318	29172	29123	30045	29414	428.6	1.46
7	High	52230	52360	52360	51402	52088	461.4	0.89	17	High	56251	56301	55949	57713	56553	788.4	1.39
	Median	42375	43720	40201	41897	42048	1453.4	3.46		Median	40106	39823	42704	42893	41382	1642.1	3.97
	Low	26714	26806	26714	26529	26691	116.2	0.44		Low	26830	27153	28445	28122	27637	768.9	2.78
8	High	51066	51950	52119	51950	51771	476.9	0.92	18	High	53262	56719	53200	53633	54203	1687.9	3.11
	Median	41121	41778	34145	36282	38332	3714.2	9.69		Median	43518	47101	43897	45407	44981	1632.1	3.63
	Low	26298	26085	26935	26978	26574	450.7	1.70		Low	28654	30114	28321	27318	28602	1157.2	4.05
9	High	53243	53113	52896	52809	53015	198.5	0.37	19	High	60839	61853	57188	58659	59635	2106.4	3.53
	Median	43642	44629	41753	43856	43470	1220.7	2.81		Median	48457	47541	47287	46930	47554	652.1	1.37
	Low	27393	27718	26880	27300	27323	345.2	1.26		Low	29543	29961	30066	30013	29896	238.9	0.80
10	High	53513	54394	52475	52371	53188	954.6	1.79	20	High	52549	53440	50165	51613	51942	1399.7	2.69
	Median	40010	40010	41199	42166	40846	1042.8	2.55		Median	37166	38913	39465	39649	38798	1132.0	2.92
	Low	26403	27503	26588	26833	26832	480.7	1.79		Low	25678	26272	28055	27643	26912	1121.5	4.17
