# Supplementary material for: Analytical Validation of a Serum Biomarker Signature for Detection of Early-Stage Pancreatic Ductal Adenocarcinoma
Source: Diagnostics (Basel). 2025 Dec 12;15(24):3177. doi: 10.3390/diagnostics15243177 (PMC12731796; doi:10.3390/diagnostics15243177)
Supplement: Supplementary file 1 [file diagnostics-15-03177-s001.zip › Supplemental Table S11.pdf]

**Supplemental Table S11. %CVs for intra-dilutional linearity measurements.**				
	**TIMP1**	**ICAM1**	**CTSD**	**THBS1**
**Dilution**	%CVs of dilutions			
1	3.9	1.5	3.6	8.4
2	1.5	1.1	3.2	2.8
3	3.3	14.6	8.7	10.7
4	2.7	6.2	0.5	13.2
5	3.4	18.7	1.5	6.2
6	4.0	24.1	5.4	4.6
7	11.1	7.7	8.8	0.9
8	6.2	14.1	4.4	1.7
9	4.0	14.8	2.0	2.0
10	-	4.7	3.2	-
Dashes indicate a 10th dilution was not run due to availability of patient samples.				
