# Supplementary material for: Analytical Validation of a Serum Biomarker Signature for Detection of Early-Stage Pancreatic Ductal Adenocarcinoma
Source: Diagnostics (Basel). 2025 Dec 12;15(24):3177. doi: 10.3390/diagnostics15243177 (PMC12731796; doi:10.3390/diagnostics15243177)
Supplement: Supplementary file 1 [file diagnostics-15-03177-s001.zip › Supplemental Table S12.pdf]

Supplemental Table S12. **Accuracy.** Average concentrations of each analyte (ng/mL) as measured by Proteomedix (PMX) and Immunovia.

TIMP 1			ICAM1			CTSD			THBS1		
Avg Conc PMX	Avg Conc Immunovia	% Bias	Avg Conc PMX	Avg Conc Immunovia	% Bias	Avg Conc PMX	Avg Conc Immunovia	% Bias	Avg Conc PMX	Avg Conc Immunovia	% Bias
448	475	6.01	183	199	9.00	415	404	-2.70	59665	60145	0.81
402	420	4.51	117	128	9.26	467	493	5.57	51136	56504	10.50
298	314	5.26	89	99	11.39	402	412	2.38	48791	50495	3.49
336	358	6.49	97	98	0.59	409	404	-1.17	40684	41377	1.70
353	360	1.88	147	160	9.00	427	434	1.61	53894	54069	0.32
387	405	4.51	178	182	2.37	327	306	-6.55	47179	49387	4.68
316	360	14.03	154	171	10.81	373	393	5.33	33785	35939	6.37
282	300	6.24	130	152	16.67	400	395	-1.32	45040	50957	13.14
311	323	3.93	211	228	8.16	407	387	-4.81	41627	42795	2.81
324	332	2.59	175	188	7.41	423	436	3.00	41024	40760	-0.64
397	402	1.25	153	177	15.84	408	418	2.40	50747	51820	2.11
366	368	0.53	149	170	13.99	442	432	-2.36	50642	52066	2.81
325	332	2.05	78	89	13.92	420	417	-0.80	43929	43473	-1.04
334	336	0.50	90	106	17.69	448	449	0.27	47902	48186	0.59
420	441	5.01	168	191	13.50	434	451	3.88	50795	52282	2.93
433	459	5.94	134	160	19.46	444	437	-1.64	54662	60485	10.65
	Avg. % Bias	**4.42**		Avg. % Bias	**11.20**		Avg. % Bias	**2.85**		Avg. % Bias	**3.80**
