# Supplementary material for: Analytical Validation of a Serum Biomarker Signature for Detection of Early-Stage Pancreatic Ductal Adenocarcinoma
Source: Diagnostics (Basel). 2025 Dec 12;15(24):3177. doi: 10.3390/diagnostics15243177 (PMC12731796; doi:10.3390/diagnostics15243177)
Supplement: Supplementary file 1 [file diagnostics-15-03177-s001.zip › Supplemental Table S2.pdf]

Supplemental Table S2. Analytical Sensitivity.			
**TIMP1**	**LOB (n=86)**	**LOD (n=78)**	**LLOQ (n=80)**
Mean absorbance	0.013	0.039	0.053
SD blank absorbance	0.005	0.007	
Limit	0.021	0.033	
Signal to noise		3.0	4.08
**ICAM1**	**LOB (n=80)**	**LOD (n=66)**	**LLOQ (n=80)**
Mean absorbance	0.019	0.087	0.079
SD blank absorbance	0.006	0.021	
Limit	0.029	0.064	
Signal to noise		4.58	4.15
**CTSD**	**LOB (n=88)**	**LOD (n=108)**	**LLOQ (n=84)**
Mean absorbance	0.035	0.092	0.189
SD blank absorbance	0.008	0.034	
Limit	0.048	0.104	
Signal to noise		2.62	5.4
**THBS1**	**LOB (n=84)**	**LOD (n=70)**	**LLOQ (n=80)**
Mean absorbance	0.006	0.132	0.168
SD blank absorbance	0.002	0.022	
Limit	0.008	0.045	
Signal to noise		22	28
