# Supplementary material for: Analytical Validation of a Serum Biomarker Signature for Detection of Early-Stage Pancreatic Ductal Adenocarcinoma
Source: Diagnostics (Basel). 2025 Dec 12;15(24):3177. doi: 10.3390/diagnostics15243177 (PMC12731796; doi:10.3390/diagnostics15243177)
Supplement: Supplementary file 1 [file diagnostics-15-03177-s001.zip › Supplemental Table S3.pdf]

Supplemental Table S3. Linearity of TIMP1 calibration samples. R^2 values for each run are listed in the top row of the middle section. A-D are the four coefficients of the fit of the linear regression curve for each run.

	Expected Conc. (ng/mL)	Run 1 Repeat	Run 2	Run 3	Run 4	Run 5	Run 6	Run 7	Run 8	Run 9	Run 10	Run 11	Run 6 Repeat	Run 12	Run 13	Run 14	Run 15	Run 12 Repeat	Run 16	Run 17	Run 18	Run 19	Run 20	Run 21	Run 22
**Date**		9/16/24	8/31/24	9/1/24	9/2/24	9/3/24	9/4/24	9/5/24	9/6/24	9/7/24	9/8/24	9/9/24	9/10/24	9/12/24	9/13/24	9/14/24	9/15/24	9/17/24	9/18/24	9/19/24	9/20/24	9/22/24	9/23/24	9/24/24	9/26/24
**Cal. 1**	7.53	7.53	7.53	7.53	7.53	7.53	7.53	7.53	7.53	7.54	7.53	7.53	7.53	7.53	7.53	7.53	7.53	7.53	7.52	7.53	7.53	7.53	7.53	7.53	7.53
**Cal. 2**	3.89	3.89	3.89	3.89	3.89	3.89	3.89	3.89	3.89	3.86	3.89	3.89	3.89	3.89	3.89	3.89	3.89	3.89	3.89	3.89	3.89	3.89	3.89	3.89	3.89
**Cal. 3**	1.68	1.69	1.69	1.68	1.68	1.69	1.68	1.69	1.69	1.74	1.67	1.67	1.68	1.68	1.68	1.69	1.68	1.69	1.67	1.69	1.69	1.69	1.68	1.69	1.70
**Cal. 4**	0.835	0.825	0.821	0.839	0.831	0.813	0.827	0.812	0.808	0.791	0.861	0.874	0.837	0.823	0.836	0.819	0.837	0.820	0.849	0.809	0.820	0.816	0.824	0.815	0.803
**Cal. 5**	0.442	0.449	0.449	0.438	0.443	0.459	0.447	0.458	0.459	0.448	0.426	0.406	0.440	0.452	0.443	0.453	0.439	0.455	0.430	0.457	0.451	0.455	0.448	0.455	0.464
	**R2 Value:**	0.9999	0.9999	1.0000	1.0000	0.9996	1.0000	0.9996	0.9995	0.9992	0.9996	0.9986	1.0000	0.9999	1.0000	0.9998	1.0000	0.9998	0.9998	0.9996	0.9999	0.9998	0.9999	0.9998	0.9993
	**A**	-0.0201	0.0003	-0.0088	-0.0086	-0.0008	-0.0011	-0.0077	-0.0256	-0.052	-0.0161	0.0232	0.0161	-0.0075	0.0036	-0.0185	-0.0129	-0.0064	-0.0003	-0.0183	-0.0217	-0.0165	-0.0117	-0.0274	-0.0107
	**B**	1.421	1.4633	1.4021	1.4298	1.5043	1.4227	1.4427	1.3774	1.0625	1.323	1.6732	1.519	1.4868	1.583	1.4356	1.5495	1.4632	1.5064	1.3865	1.387	1.4206	1.4901	1.378	1.5122
	**C**	8.2848	7.9133	8.775	7.8662	7.0199	7.6438	7.7408	8.7974	92.873	9.8886	5.8214	6.6304	7.6994	5.3991	6.9967	6.3659	8.2297	8.8959	8.6524	7.7102	8.8692	6.7696	8.2443	6.409
	**D**	4.8088	3.9282	3.848	3.6928	3.5864	4.661	4.002	4.3715	32.0145	4.8758	2.7025	3.7622	3.8034	3.471	3.793	3.3088	4.0205	4.7227	4.6456	4.309	4.7025	4.2124	4.4381	3.481
	**Accuracy (%)**																								
	**Cal. 1**	100	100	100	100	100	100	100	100	100	100	100	100	100	100	100	100	100	100	100	100	100	100	100	100
	**Cal. 2**	100	100	100	100	100	100	100	100	99.1	100	100	100	100	100	100	100	100	100	100	100	100	100	100	100
	**Cal. 3**	100	100	100	100	101	100	101	101	104	99.2	99.1	100	100	100	100	100	100	99.4	101	100	101	100	101	101
	**Cal. 4**	98.9	98.4	100	100	97.4	99.0	97.2	96.8	94.7	103	105	100	98.6	100	98.1	100	98.3	102	96.9	98.2	97.7	98.7	97.7	96.2
	**Cal. 5**	102	102	99.1	100	104	101	104	104	101	96.3	91.9	100	102	100	103	99.4	103	97.4	103	102	103	101	103	105
