# Supplementary material for: Analytical Validation of a Serum Biomarker Signature for Detection of Early-Stage Pancreatic Ductal Adenocarcinoma
Source: Diagnostics (Basel). 2025 Dec 12;15(24):3177. doi: 10.3390/diagnostics15243177 (PMC12731796; doi:10.3390/diagnostics15243177)
Supplement: Supplementary file 1 [file diagnostics-15-03177-s001.zip › Supplemental Table S4.pdf]

Supplemental Table S4. Linearity of ICAM1 calibration samples. R^2 values for each run are listed in the top row of the middle section. A-D are the four coefficients of the fit of the linear regression curve for each run.

	Expected Conc. (ng/mL)	Run 1	Run 2	Run 3	Run 4	Run 5	Run 6	Run 7	Run 8	Run 9	Run 10	Run 11	Run 6 Repeat	Run 12	Run 13	Run 14	Run 15	Run 16	Run 17 Repeat	Run 18 Repeat	Run 19	Run 20	Run 21	Run 22
Date		8/29/24	8/31/24	9/1/24	9/2/24	9/3/24	9/4/24	9/5/24	9/6/24	9/7/24	9/8/24	9/9/24	9/10/24	9/12/24	9/13/24	9/14/24	9/15/24	9/16/24	9/24/24	9/25/24	9/20/24	9/22/24	9/23/24	9/26/24
Cal. 1	7.76	7.76	7.76	7.76	7.76	7.76	7.76	7.76	7.77	7.77	7.76	7.77	7.76	7.76	7.76	7.76	7.77	7.77	7.76	7.78	7.76	7.76	7.76	7.76
Cal. 2	4.74	4.74	4.74	4.73	4.74	4.74	4.74	4.73	4.75	4.73	4.73	4.73	4.74	4.74	4.74	4.74	4.74	4.73	4.73	4.68	4.72	4.74	4.74	4.74
Cal. 3	2.71	2.71	2.74	2.76	2.73	2.73	2.71	2.76	2.76	2.77	2.78	2.74	2.71	2.74	2.72	2.72	2.74	2.79	2.79	2.72	2.81	2.73	2.73	2.74
Cal. 4	1.82	1.82	1.77	1.72	1.79	1.78	1.83	1.73	1.75	1.71	1.74	1.74	1.83	1.77	1.78	1.80	1.77	1.74	1.70	1.68	1.71	1.79	1.79	1.76
Cal. 5	0.692	0.689	0.729	0.765	0.721	0.737	0.677	0.765	0.802	0.777	0.705	0.778	0.652	0.754	0.737	0.715	0.734	0.714	0.764	0.491	0.697	0.723	0.724	0.768
	R2 Value:	1.0000	0.9993	0.9972	0.9995	0.9993	1.0000	0.9975	0.9970	0.9965	0.9992	0.9971	0.9999	0.9988	0.9993	0.9997	0.9991	0.9991	0.9967	0.9908	0.9985	0.9995	0.9996	0.9982
	A	0.0435	0.0714	0.067	0.0535	0.0839	0.0919	0.0743	0.0447	0.0599	0.0609	0.0582	0.0546	0.1083	0.0485	0.0655	0.0706	0.0589	0.0649	0.0645	0.067	0.0685	0.0929	0.0591
	B	2.1309	1.9183	2.014	2.0909	2.063	2.1037	2.0082	2.2106	2.026	1.9999	2.1478	2.4551	2.2207	2.3681	2.3515	2.0189	2.1755	2.1017	2.0175	2.0157	2.1969	2.1427	2.1752
	C	17.9127	30.2633	17.9218	19.0106	14.5481	11.9063	17.5044	15.7016	19.7721	28.2743	13.9884	8.993	9.5675	9.6211	9.6622	22.2681	20.6427	27.3592	37.6913	34.1993	11.8871	11.8535	12.764
	D	8.3441	28.1735	11.5509	11.7635	10.2591	6.385	10.1497	7.1903	14.5826	24.3511	6.6627	3.1347	6.3936	3.1294	3.954	18.1772	13.9079	27.9299	41.438	38.5058	5.9458	7.1509	7.7064
	Accuracy (%)																							
	Cal. 1	100	100	100	100	100	100	100	100	100	100	100	100	100	100	100	100	100	100	100	100	100	100	100
	Cal. 2	100	100	100	100	100	100	100	100	100	100	100	100	100	100	100	100	100	100	98.7	100	100	100	100
	Cal. 3	100	101	102	101	101	100	102	102	102	102	101	100	101	100	100	101	103	103	101	104	101	101	101
	Cal. 4	100	97.2	94.8	98.3	97.6	100	95.1	96.1	93.9	95.8	95.3	100	97.1	98.1	98.7	97.3	95.6	93.3	92.2	94.0	98.2	98.3	96.5
	Cal. 5	100	105	111	104	107	97.9	110	116	112	102	112	94.2	109	106	103	106	103	110	70.9	101	104	105	111
