# Supplementary material for: Analytical Validation of a Serum Biomarker Signature for Detection of Early-Stage Pancreatic Ductal Adenocarcinoma
Source: Diagnostics (Basel). 2025 Dec 12;15(24):3177. doi: 10.3390/diagnostics15243177 (PMC12731796; doi:10.3390/diagnostics15243177)
Supplement: Supplementary file 1 [file diagnostics-15-03177-s001.zip › Supplemental Table S5.pdf]

Supplemental Table S5. Linearity of CTSD calibration samples. R^2 values for each run are listed in the top row of the middle section. A-D are the four coefficients of the fit of the linear regression curve for each run.

	Expected Conc. (ng/mL)	Run 1	Run 2	Run 3 Repeat	Run 4	Run 5	Run 6	Run 7	Run 8	Run 9	Run 10	Run 11	Run 6 Repeat	Run 12 Repeat	Run 13	Run 14	Run 15	Run 16	Run 17	Run 18	Run 19	Run 19 Repeat	Run 20 Repeat	Run 21	Run 22
Date		8/29/24	8/31/24	9/24/24	9/2/24	9/3/24	9/4/24	9/5/24	9/6/24	9/7/24	9/8/24	9/9/24	9/10/24	9/17/24	9/13/24	9/14/24	9/15/24	9/16/24	9/18/24	9/19/24	9/20/24	9/25/24	9/25/24	9/23/24	9/26/24
Cal. E	18.8	18.8	18.9	18.9	18.8	18.8	18.9	18.9	18.9	18.9	18.8	18.8	18.9	18.9	*18.8	18.9	18.8	18.9	18.9	18.9	18.9	18.8	18.8	18.8	18.9
Cal. D	15.3	15.4	15.0	15.1	15.4	*15.2	15.1	15.2	15.2	14.8	15.3	15.2	14.8	15.0	15.4	15.1	15.3	15.0	15.1	15.1	14.8	15.4	15.3	15.3	15.1
Cal. C	12.3	12.0	12.6	12.4	12.1	12.4	12.5	12.3	12.2	12.4	12.2	12.3	12.3	12.6	12.2	12.5	12.3	12.5	12.6	12.5	12.3	12.2	12.3	12.4	12.4
Cal. B	8.63	8.79	8.39	8.74	8.79	8.60	8.64	8.51	8.88	8.57	8.73	8.75	8.71	8.80	8.74	8.60	8.66	8.68	8.49	8.66	8.70	8.73	8.69	8.59	8.84
Cal. A	4.78	4.69	4.88	4.66	4.64	4.75	4.68	4.76	4.57	4.56	4.70	4.65	4.30	4.34	4.73	4.71	4.76	4.69	4.79	4.68	*4.34	4.74	4.72	4.80	4.58
*Masked one value due to outlier status																									
	R2 Value:	0.9991	0.9981	0.9994	0.9991	0.9998	0.9995	0.9998	0.9983	0.9990	0.9998	0.9995	0.9964	0.9964	0.9997	0.9995	1.0000	0.9994	0.9992	0.9995	0.9971	0.9997	0.9999	0.9999	0.9984
	A	0.0277	-0.0093	0.0871	0.0949	0.0313	0.0595	0.0018	0.0807	0.1066	0.0129	0.0631	0.1586	0.1203	0.1336	0.0449	0.0232	0.0311	0.0282	0.0772	0.1432	0.0568	0.0133	0.0395	0.0814
	B	1.8381	1.9886	2.0404	2.8365	1.9739	2.0468	1.7992	2.0316	2.2568	1.9418	2.0374	2.3534	2.4171	2.1908	2.1311	1.9783	2.0101	2.102	2.0169	2.3347	1.9738	1.8975	2.038	1.9306
	C	58.0911	28.3402	32.8106	18.4685	85.307	88.1363	81.2028	36.1619	24.237	71.8945	76.5165	27.0679	46.386	28.2696	55.8088	54.8496	31.9460	51.5572	88.2495	26.5339	27.7612	78.207	25.1626	40.8641
	D	14.7045	5.235	9.5096	3.5067	32.7733	46.2913	30.5075	11.0255	6.0947	23.2717	38.7394	7.519	17.6997	8.0883	21.0583	16.0632	7.9968	14.2432	50.4733	8.036	7.6027	30.5262	6.4314	12.4366
	Accuracy (%)																								
	Cal. 1	100	100	100	100	100	100	100	100	101	100	100	100	100	100	101	100	101	100	100	100	100	100	100	101
	Cal. 2	101	98.3	98.8	101	99.2	98.7	100	99.2	97.0	100	99.5	96.6	98.1	101	98.7	100	98.3	98.7	98.8	96.8	101	100	100	98.4
	Cal. 3	97.9	103	101	98.5	101	102	100	100	101	100	100	100	102	98.9	102	100	101	102	101	100	98.8	100	100	101
	Cal. 4	102	97.3	101	102	100	100	98.6	103	99.3	101	101	101	102	101	100	100	101	98.4	100	101	101	101	100	102
	Cal. 5	98.1	102	97.5	97.1	99.4	98.0	100	95.6	95.4	98.4	97.4	90.0	90.9	98.9	98.5	100	98.1	100	97.8	90.9	99.2	98.8	100	95.8
