# Supplementary material for: Analytical Validation of a Serum Biomarker Signature for Detection of Early-Stage Pancreatic Ductal Adenocarcinoma
Source: Diagnostics (Basel). 2025 Dec 12;15(24):3177. doi: 10.3390/diagnostics15243177 (PMC12731796; doi:10.3390/diagnostics15243177)
Supplement: Supplementary file 1 [file diagnostics-15-03177-s001.zip › Supplemental Table S6.pdf]

Supplemental Table S6. Linearity of THBS1 calibration samples. R^2 values for each run are listed in the top row of the middle section. A-D are the four coefficients of the fit of the linear regression curve for each run.

	Expected Conc. (ng/mL)	Run 1 Repeat	Run 2	Run 3	Run 4	Run 5	Run 6	Run 7	Run 8	Run 9	Run 10	Run 11	Run 6 Repeat	Run 12	Run 13	Run 14	Run 15	Run 16	Run 12 Repeat	Run 17	Run 18	Run 19	Run 20	Run 21	Run 22
Date		9/24/24	8/31/24	9/1/24	9/2/24	9/3/24	9/4/24	9/5/24	9/6/24	9/7/24	9/8/24	9/9/24	9/10/24	9/12/24	9/13/24	9/14/24	9/15/24	9/16/24	9/17/24	9/18/24	9/19/24	9/20/24	9/22/24	9/23/24	9/26/24
Cal. E	71.6	71.7	71.6	71.5	71.5	71.6	71.5	71.5	71.5	71.5	71.5	71.5	71.9	72.0	71.5	72.2	71.9	71.7	71.8	72.1	72.2	71.9	71.5	71.7	71.7
Cal. D	44.0	44.0	44.0	43.3	44.2	44.0	43.4	44.2	43.3	44.3	44.3	44.2	43.4	43.4	44.3	42.6	43.2	43.8	43.9	42.8	42.6	42.9	44.0	44.0	43.8
Cal. C	25.4	24.6	25.4	24.9	25.1	25.3	24.7	25.1	24.9	24.8	24.7	25.0	25.4	25.5	24.8	26.4	25.8	25.1	25.4	26.6	26.8	26.8	24.3	24.5	*19.6
Cal. B	14.7	16.0	*20.8	15.1	15.0	14.8	15.4	15.1	15.2	15.3	15.5	15.1	16.1	15.6	15.4	15.0	15.1	15.3	16.1	14.2	14.1	13.6	16.3	16.1	15.0
Cal. A	7.03	6.39	7.03	6.31	6.85	6.96	6.15	6.83	6.36	6.73	6.61	6.86	5.68	6.12	6.64	6.50	6.65	6.72	6.22	6.87	6.96	7.34	6.18	6.32	6.86
	*Data point was outside acceptance criteria and was excluded																								
	R2 Value:	0.9958	1.0000	0.9982	0.9997	1.0000	0.9967	0.9995	0.9979	0.9989	0.9982	0.9995	0.9903	0.9958	0.9985	0.9980	0.9990	0.9991	0.9943	0.9988	0.9984	0.9965	0.9931	0.9947	0.9997
	A	0.0219	0.0701	0.0833	0.0718	0.0403	0.0696	0.067	0.0907	0.0694	0.0933	0.0422	0.0845	0.0916	0.0958	0.0507	-0.0022	-0.0303	0.0862	0.0542	0.0505	0.0056	0.0172	0.051	-0.0332
	B	1.1245	1.6018	1.5449	1.682	1.4709	1.5187	1.6241	1.4283	1.4766	1.6055	1.3671	1.5515	1.5996	1.6294	1.3095	1.1288	1.2131	1.5397	1.5387	1.3971	1.2579	1.2016	1.1468	1.0884
	C	361.1783	70.7107	80.3479	53.226	73.0596	84.2278	60.3547	91.6644	70.2173	66.1442	92.5754	94.9029	78.7923	63.7507	112.7958	206.4693	111.6931	95.2588	86.1514	92.9078	94.9685	375.8692	365.4036	298.1581
	D	13.4726	3.6609	4.1471	2.7551	3.4209	5.2218	3.1112	5.8839	4.7892	4.3451	6.1021	5.1601	3.6329	4.0881	6.3973	10.857	6.2629	4.9568	4.3379	5.4252	5.3318	15.4737	17.0486	14.1181
	Accuracy (%)																								
	Cal. 1	100	100	100	100	100	100	100	100	100	100	100	100	101	100	101	100	100	100	101	101	100	100	100	100
	Cal. 2	100	100	98.4	100	100	98.6	100	98.4	101	101	101	98.5	98.6	101	96.8	98.1	100	100	97.4	96.9	97.5	100	100	100
	Cal. 3	97.0	100	97.9	98.9	99.5	97.3	98.7	98.1	97.6	97.4	98.3	100	100	97.7	104	102	98.7	100	105	106	105	95.6	96.6	*77.0
	Cal. 4	109	*141	103	102	101	104	103	104	104	105	103	109	106	105	102	102	104	110	96.6	96.1	92.4	111	110	102
	Cal. 5	91.0	100	89.7	97.5	99.0	87.4	97.2	90.4	95.8	94.0	97.5	80.8	87.0	94.5	92.5	94.6	95.6	88.5	97.8	98.9	104	88.0	89.9	97.5
