# Supplementary material for: Analytical Validation of a Serum Biomarker Signature for Detection of Early-Stage Pancreatic Ductal Adenocarcinoma
Source: Diagnostics (Basel). 2025 Dec 12;15(24):3177. doi: 10.3390/diagnostics15243177 (PMC12731796; doi:10.3390/diagnostics15243177)
Supplement: Supplementary file 1 [file diagnostics-15-03177-s001.zip › Supplemental Table S7.pdf]

Supplemental Table S7. TIMP1 Precision. Concentrations and %CVs for individual measurements of TIMP1.

Run ID	Concentration Level	Concentration (ng/mL) * dilution factor (164)				Intra-day Average	Intra-day SD	Intra-day %CV	Run ID	Concentration Level	Concentration (ng/mL) * dilution factor (164)				Intra-day Average	Intra -day SD	Intra-day %CV
1	High	860	845	834	828	842	13.9	1.65	13	High	853	846	857	834	848	10.0	1.18
	Median	354	315	333	307	327	21.1	6.45		Median	346	339	339	337	340	4.2	1.25
	Low	154	150	165	161	157	6.9	4.37		Low	152	146	153	151	151	3.4	2.26
2	High	827	827	829	794	819	17.0	2.08	14	High	877	874	824	878	863	26.2	3.03
	Median	349	337	336	326	337	9.4	2.79		Median	342	321	330	356	337	14.9	4.41
	Low	156	149	165	161	158	7.2	4.54		Low	162	159	170	90	145	37.3	25.61
3	High	797	796	854	926	843	61.6	7.31	15	High	792	781	828	793	798	20.3	2.55
	Median	326	304	351	348	332	22.0	6.61		Median	310	302	285	297	298	10.2	3.42
	Low	155	158	162	161	159	3.2	2.02		Low	146	141	122	120	132	13.3	10.02
4	High	837	830	855	837	840	10.6	1.27	16	High	873	865	843	845	856	14.5	1.70
	Median	323	324	345	339	333	11.0	3.30		Median	365	352	374	371	366	10.0	2.73
	Low	156	154	160	156	157	2.2	1.40		Low	178	177	186	181	180	3.8	2.11
5	High	823	834	841	824	831	8.5	1.02	17	High	891	854	849	782	844	45.7	5.41
	Median	328	323	319	319	322	4.4	1.37		Median	327	353	336	327	336	12.6	3.74
	Low	158	158	170	165	163	5.8	3.55		Low	174	161	156	153	161	9.0	5.62
6	High	354	337	758	743	548	234.0	42.70	18	High	860	829	870	844	851	18.3	2.15
	Median	305	303	284	272	291	15.8	5.43		Median	322	314	332	324	323	7.4	2.28
	Low	115	110	140	138	126	15.5	12.29		Low	158	159	164	157	159	3.3	2.04
7	High	1059	879	860	788	897	115.3	12.87	19	High	829	799	830	804	815	16.4	2.01
	Median	347	332	319	355	338	16.2	4.80		Median	342	339	322	316	330	12.6	3.84
	Low	159	160	159	157	159	1.3	0.79		Low	162	155	155	158	157	3.7	2.36
8	High	814	835	820	729	800	47.9	6.00	20	High	855	832	829	814	832	17.2	2.06
	Median	328	318	324	308	319	8.5	2.67		Median	344	344	338	337	341	3.7	1.07
	Low	157	142	154	151	151	6.3	4.19		Low	169	161	160	153	161	6.4	4.00
9	High	813	800	901	864	845	46.7	5.52	21	High	834	820	834	834	831	7.1	0.86
	Median	370	362	357	343	358	11.4	3.18		Median	335	321	326	325	327	6.2	1.89
	Low	155	154	157	153	155	1.6	1.03		Low	169	158	156	150	158	7.7	4.88
10	High	824	807	845	802	820	19.3	2.35	22	High	725	847	790	804	792	50.4	6.37
	Median	314	296	326	315	313	12.3	3.92		Median	331	327	326	317	325	5.7	1.76
	Low	141	134	150	146	143	7.0	4.87		Low	170	165	165	164	166	2.6	1.54
11	High	834	807	848	808	824	20.3	2.46	23	High	778	827	776	798	795	23.9	3.01
	Median	308	289	312	305	303	9.9	3.27		Median	329	311	320	320	320	7.3	2.29
	Low	147	144	145	134	142	6.0	4.20		Low	161	162	156	154	158	3.6	2.26

12	High	129	123	115	111	119	7.9	6.63	
	Median	125	122	86	81	103	23.2	22.41	
	Low	43	41	70	70	56	15.9	28.57	
