# Supplementary material for: Analytical Validation of a Serum Biomarker Signature for Detection of Early-Stage Pancreatic Ductal Adenocarcinoma
Source: Diagnostics (Basel). 2025 Dec 12;15(24):3177. doi: 10.3390/diagnostics15243177 (PMC12731796; doi:10.3390/diagnostics15243177)
Supplement: Supplementary file 1 [file diagnostics-15-03177-s001.zip › Supplemental Table S8.pdf]

Supplemental Table S8. ICAM1 Precision. Concentrations and %CVs for individual measurements of ICAM1.

Run ID	Concentration Level	Concentration (ng/mL) * dilution factor (41)				Intra-day Average	Intra-day SD	Intra-day %CV	Run ID	Concentration Level	Concentration (ng/mL) * dilution factor (41)				Intra-day Average	Intra-day SD	Intra-day %CV
	High	254	259	278	257	262	10.7	4.07		High	313	291	273	268	286	20.5	7.14
1	Median	163	154	161	148	156	7.0	4.50	12	Median	158	170	135	133	149	17.9	11.98
	Low	119	111	118	113	115	3.7	3.23		Low	116	114	114	83.4	107	15.6	14.62
	High	285	274	286	291	284	7.1	2.51		High	305	297	268	287	289	15.9	5.51
2	Median	167	156	157	160	160	4.7	2.95	13	Median	180	175	150	151	164	15.7	9.54
	Low	121	126	131	132	127	5.1	4.05		Low	130	129	136	133	132	3.2	2.44
	High	272	287	285	305	287	13.3	4.63		High	272	239	286	244	260	22.4	8.59
3	Median	163	164	161	169	164	3.2	1.95	14	Median	163	164	152	146	156	8.5	5.46
	Low	121	130	124	129	126	4.4	3.49		Low	127	121	108	97	114	13.5	11.86
	High	297	296	264	265	281	18.5	6.61		High	277	283	257	265	271	11.5	4.25
4	Median	175	173	168	162	169	6.0	3.52	15	Median	149	143	138	100	132	22.4	16.93
	Low	120	117	126	122	121	3.7	3.06		Low	127	90.0	128	131	119	19.6	16.43
	High	281	270	269	279	274	5.9	2.15		High	292	274	304	307	294	15.0	5.10
5	Median	157	149	150	148	151	4.3	2.84	16	Median	163	151	133	140	147	13.2	9.01
	Low	119	120	124	121	121	2.2	1.80		Low	124	130	122	123	125	3.8	3.06
	High	309	316	267	262	289	28.1	9.73		High	306	290	246	259	275	27.5	10.01
6	Median	178	172	151	150	163	14.4	8.84	17	Median	138	138	141	145	141	3.0	2.14
	Low	123	127	126	125	125	1.7	1.35		Low	120	113	132	124	122	8.1	6.64
	High	260	264	257	271	263	5.7	2.19		High	302	304	273	275	288	16.8	5.83
7	Median	149	159	150	155	153	4.8	3.16	18	Median	149	149	148	147	148	0.9	0.58
	Low	141	143	119	121	131	12.7	9.73		Low	111	116	129	130	121	9.5	7.86
	High	277	273	259	263	268	8.2	3.05		High	276	290	256	262	271	15.2	5.60
8	Median	163	157	147	151	154	7.3	4.72	19	Median	154	139	148	153	148	7.1	4.77
	Low	122	124	119	113	120	4.7	3.91		Low	79.0	90.8	124	87.4	95	19.9	20.85
	High	280	276	265	271	273	6.6	2.42		High	265	245	278	271	265	14.5	5.46
9	Median	166	167	158	155	161	5.5	3.43	20	Median	123	135	150	152	140	13.5	9.63
	Low	126	127	119	123	124	3.4	2.73		Low	119	120	121	79.5	110	20.2	18.41
	High	271	273	286	282	278	7.3	2.64		High	278	282	267	284	278	7.6	2.74
10	Median	143	139	145	144	143	2.6	1.85	21	Median	150	121	149	126	136	15.0	11.01
	Low	116	122	115	116	118	3.2	2.75		Low	115	122	122	97.1	114	11.7	10.27
	High	293	249	267	355	291	46.6	16.02									
11	Median	147	153	192	132	156	25.6	16.42									
	Low	112	149	149	147	139	18.3	13.11									
