# Supplementary material for: Analytical Validation of a Serum Biomarker Signature for Detection of Early-Stage Pancreatic Ductal Adenocarcinoma
Source: Diagnostics (Basel). 2025 Dec 12;15(24):3177. doi: 10.3390/diagnostics15243177 (PMC12731796; doi:10.3390/diagnostics15243177)
Supplement: Supplementary file 1 [file diagnostics-15-03177-s001.zip › Supplemental Table S9.pdf]

Supplemental Table S9. CTSD Precision. Concentrations and %CVs for individual measurements of CTSD. Outliers were omitted from analysis.

Run ID	Concentration Level	Concentration (ng/mL) * dilution factor (41)				Intra-day Average	Intra-day SD	Intra-day %CV	Run ID	Concentration Level	Concentration (ng/mL) * dilution factor (41)				Intra-day Average	Intra-day SD	Intra-day %CV
1	High	445	434	475	389	436	35.5	8.16	12	High	468	476	458	472	468	7.9	1.69
	Median	473	442	407	400	430	33.9	7.88		Median	470	505	462	369	452	57.9	12.81
	Low	259	250	242	222	243	15.6	6.42		Low	291	310	278	271	287	17.5	6.07
2	High	456	444	443	427	442	11.9	2.69	13	High	448	457	447	471	456	11.1	2.43
	Median	441	438	389	395	416	27.9	6.72		Median	463	468	406	Outlier	446	34.2	7.68
	Low	260	265	256	242	256	10.1	3.95		Low	275	278	263	258	269	9.6	3.57
3	High	473	481	435	438	457	23.8	5.21	14	High	460	459	450	459	457	4.9	1.06
	Median	438	456	493	421	452	30.7	6.80		Median	448	470	Outlier	472	463	13.5	2.91
	Low	236	245	273	264	255	17.2	6.76		Low	238	232	209	146	206	41.7	20.24
4	High	470	469	439	448	456	15.7	3.43	15	High	433	443	469	480	456	21.9	4.81
	Median	460	474	456	445	459	12.0	2.61		Median	444	454	462	475	459	13.4	2.92
	Low	288	290	272	344	299	31.1	10.41		Low	282	301	259	258	275	20.8	7.57
5	High	458	454	448	456	454	4.4	0.97	16	High	470	501	445	456	468	24.0	5.12
	Median	464	456	459	415	448	22.7	5.07		Median	515	504	431	426	469	47.0	10.02
	Low	295	308	270	252	281	24.7	8.79		Low	303	318	266	267	288	26.0	9.00
6	High	433	440	436	450	440	7.5	1.71	17	High	477	484	482	465	477	8.3	1.73
	Median	458	466	402	408	434	33.2	7.66		Median	463	486	446	452	462	17.6	3.82
	Low	285	294	257	251	272	21.1	7.78		Low	266	250	281	263	265	12.9	4.86
7	High	420	418	427	424	422	3.9	0.93	18	High	442	439	488	463	458	22.4	4.90
	Median	452	435	396	386	417	31.3	7.49		Median	460	456	435	467	455	13.9	3.06
	Low	267	267	233	226	248	21.6	8.70		Low	252	249	278	267	262	13.6	5.20
8	High	440	437	465	447	447	12.5	2.79	19	High	448	475	451	462	459	11.9	2.59
	Median	466	463	437	429	449	18.5	4.13		Median	452	464	431	433	445	15.8	3.55
	Low	275	281	253	247	264	16.4	6.21		Low	264	269	274	293	275	12.9	4.69
9	High	482	489	471	474	479	8.1	1.69	20	High	450	456	445	444	449	5.2	1.17
	Median	463	472	436	420	448	23.8	5.32		Median	458	455	460	442	454	8.2	1.80
	Low	280	299	267	267	278	15.2	5.46		Low	259	252	281	251	261	14.1	5.40
10	High	424	413	425	419	420	5.6	1.34	21	High	453	445	468	467	458	11.4	2.48
	Median	437	439	434	444	438	4.1	0.93		Median	500	494	445	445	471	30.1	6.40
	Low	235	230	230	219	229	6.8	2.96		Low	275	279	269	265	272	6.1	2.26
11	High	464	481	474	481	475	7.9	1.67	22	High	469	479	467	496	478	13.3	2.77
	Median	512	497	448	489	486	27.4	5.64		Median	483	492	446	446	467	24.2	5.19

	Low	281	286	255	244	267	20.2	7.59		Low	288	302	274	288	288	11.4	3.97
